# Decreased miR-198 expression and its prognostic significance in human gastric cancer

**DOI:** 10.1186/s12957-016-0784-x

**Published:** 2016-02-06

**Authors:** Zhigang Cui, Xin Zheng, Di Kong

**Affiliations:** Department of Oncology, Tianjin Nankai Hospital, No.6 of Changjiang Road, Nankai District, Tianjin, 300100 People’s Republic of China

**Keywords:** miR-198, Gastric cancer, Prognosis, Overall survival

## Abstract

**Background:**

MicroRNAs (miRNAs) have been proved to play important roles in the tumorigenesis and development of human gastric cancer (GC). Our study aims to investigate the expression and clinical significance of miR-198 in GC patients.

**Methods:**

Quantitative real-time polymerase chain reaction (RT-PCR) was performed to evaluate miR-198 levels in 106 pairs of GC specimens and adjacent noncancerous tissues. Then, the associations of miR-198 expression with clinicopathological factors and patient’s survival were determined.

**Results:**

The expression levels of miR-198 in GC tissues were significantly lower than those in corresponding noncancerous tissues (*p* < 0.01). Decreased miR-198 expression was significantly associated with larger tumor size, deeper invasion depth, positive lymph node metastasis, advanced tumor-node-metastasis (TNM) stage, and shorter overall survival. Moreover, multivariate regression analysis identified low miR-198 expression as an independent predictor of poor survival.

**Conclusions:**

These findings suggested that miR-198 downregulation may be associated with progression of GC and that this miR may be an independent prognostic marker for GC patients.

## Background

Gastric cancer (GC) is the fourth most prevalent human malignancy and the second leading cause of cancer death worldwide [1]. The majority of GC patients are diagnosed at advanced stage due to vague initial symptoms [2]. Despite recent advances in surgical techniques, new chemotherapy regimens, radiotherapy, and molecular targeted therapy, the clinical outcome of patients with GC remains dismal, with a 5-year survival rate of 25 % or less [3]. Previous studies have reported many oncogenes and tumor suppressor genes closely associated with GC [4–6], but the highly complex molecular mechanisms underlying its carcinogenesis and progression are still obscure. Therefore, it is urgent to identify reliable biomarkers of GC for its early diagnosis, effective therapy, and prognosis evaluation.

MicroRNAs (miRNAs) are a class of naturally occurring, short (about 22 nucleotides in length), single-stranded, non-protein-coding RNAs that negatively regulate gene expression [7]. It is estimated that about 60 % genes can be regulated by miRNAs [8]. They suppress translation or promote the degradation of target messenger RNAs (mRNAs) through base pairing with the 3ʹ-untranslated regions (3ʹ-UTRs) [7, 9]. Previous research has shown that miRNAs have critical roles in various biological processes, such as development, differentiation, cell growth, inflammation, stress response, and endocrine homeostasis [10]. Emerging evidence demonstrates that aberrant miR expression is highly associated with cancer initiation and progression, which may provide a new but promising way to deal with cancer [11–13]. miRNAs can function as either oncogenes or tumor suppressors according to the roles of their target genes. Deregulation or dysfunction of miRNAs is involved in many processes of tumor progression including cell proliferation, apoptosis, invasion, metastasis, angiogenesis, and epithelial-to-mesenchymal transition [14–16]. Functional miRNAs may be applied for cancer diagnosis and prognosis and also act as potential novel therapeutic targets.

miR-198 is a recently identified cancer-related miR. It is observed to be downregulated in lung cancer [17], colorectal cancer [18], hepatocellular carcinoma [19], pancreatic cancer [20], ovarian cancer [21], and prostate cancer [22], and acts as a potential tumor suppressor in these tumors. Yet miR-198 was reported to be upregulated in retinoblastoma and squamous cell carcinoma of the tongue [23, 24], indicating that miR-198 may not behave as a tumor suppressor in all cases. However, the expression and clinical significance of miR-198 in GC is still unclear. In the present study, we examined miR-198 expression in GC tissues and paired adjacent noncancerous tissues. The relationship between miR-198 expression and clinicopathological features and patient’s survival was also analyzed.

## Methods

### Patients and clinical specimens

This study was approved by the Research Ethics Committee of Tianjin Nankai Hospital. Written informed consent was obtained from all of the patients. All specimens were handled and made anonymous according to the ethical and legal standards.

Fresh primary GC tissues and matched normal adjacent tissues (≥3 cm away from tumor margin) were obtained from 106 consecutive patients who received radical excision at Tianjin Nankai Hospital between February 2007 and June 2010. The diagnosis of all samples was histopathologically confirmed by two pathologists. All specimens were frozen immediately in liquid nitrogen and stored at −80 °C until analysis. Patients with two or more different malignancies were excluded. None of the patients had received preoperative radiotherapy or chemotherapy. Patient characteristics are shown in Table 1. Tumors were staged according to the tumor-node-metastasis (TNM) staging system of the International Union Against Cancer. Follow-up data were available for all patients. Overall survival was defined as the amount of time from the day of primary surgery to the date of death or the end of follow-up (for living patients).

**Table 1:** Correlation between miR-198 expression and different clinicopathological features in gastric cancer patients

	Low miR-198 expression	High miR-198 expression	*p* value
Age			
≥60	36 (48.0 %)	39 (52.0 %)	
<60	17 (54.8 %)	14 (45.2 %)	0.336
Gender			
Male	28 (46.7 %)	32 (53.3 %)	
Female	25 (54.3 %)	21 (45.7 %)	0.278
Differentiation			
Well-moderate	14 (41.2 %)	20 (58.8 %)	
Poor	39 (54.2 %)	33 (45.8 %)	0.149
Lauren type			
Intestinal	29 (43.9 %)	37 (56.1 %)	
Diffuse and mixed	24 (60.0 %)	16 (40.0 %)	0.08
Tumor size			
≥5 cm	38 (60.3 %)	25 (39.7 %)	
<5 cm	15 (34.9 %)	28 (65.1 %)	0.009
Invasion depth			
T1, T2	16 (34.8 %)	30 (65.2 %)	
T3, T4	37 (61.7 %)	23 (38.3 %)	0.005
TNM stage			
I/II	13 (35.1 %)	24 (64.9 %)	0.02
III	40 (58.0 %)	29 (42.0 %)	
Lymphatic metastasis			
Negative	12 (35.3 %)	22 (64.7 %)	0.03
Positive	41 (56.9 %)	31 (43.1 %)	

### RNA extraction and quantitative real-time polymerase chain reaction (PCR)

Total RNA was extracted from clinical specimens by using Trizol reagent (Invitrogen Corp, Carlsbad, CA, USA) according to the manufacturer’s instructions. RNA concentration was measured using a NanoDrop ND-1000 spectrophotometer (Thermo Scientific, Wilmington, DE). Complementary DNA (cDNA) was synthesized from isolated RNA using TaqMan MicroRNA Reverse Transcription Kit and miRNA-specific stem-loop primers (both from Applied Biosystems, Foster City, CA, USA). Real-time PCR was performed with a Taqman MicroRNA Assay Kit (Applied Biosystems) on ABI7500 real-time PCR detection system. Quantitative PCR was conducted at 95 °C for 10 min followed by 40 cycles of 95 °C for 15 s and 60 °C for 60 s. U6 small nuclear RNA was used as an internal control. All reactions were run in triplicates. The cycle threshold (CT) values were recorded, and the relative amount of miR-198 to U6 was calculated using the equation 2^−ΔCT^, where ΔCT = (CT_miR-198_ − CT_U6_).

### Statistics

All statistical analyses were performed using the SPSS 16.0 software package (SPSS, Chicago, IL, USA). The significance of differences between groups was estimated by Student’s *t* test and Chi-square test. Survival curves were constructed with the Kaplan-Meier method and compared by log-rank test. The significance of survival variables was evaluated using a multivariate Cox proportional hazards regression analysis. *p* < 0.05 was considered statistically significant.

## Results

### Downregulation of miR-198 in human GC tissues

**Fig. 1:**
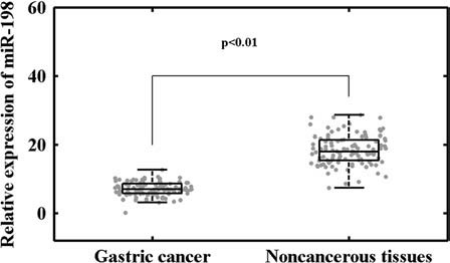
miR-198 expression was significantly lower in gastric cancer samples than in the corresponding noncancerous tissues (*p* < 0.01, paired *t* test). miR-198 expression levels were calculated by the 2^−ΔCT^ method and normalized to U6 small nuclear RNA

miR-198 expression was detected in 106 pairs of GC and corresponding adjacent noncancerous tissues normalized to U6 small nuclear RNA. As shown in Fig. 1, miR-198 expression in cancer tissues was distinctly decreased compared to noncancerous tissues. The results showed that the relative level of miR-198 expression in GC samples (mean ± SD 7.05 ± 1.98) was significantly lower than that in corresponding adjacent normal tissues (mean ± SD 18.38 ± 4.30; *p* < 0.01).

### Correlation between miR-198 expression and clinical features of GC

We further analyzed the association between miR-198 expression levels and clinicopathological characteristics of GC. GC samples were classified into low miR-198 expression group (*n* = 53) and high miR-198 expression group (*n* = 53) according to the median miR-198 expression level (relative to U6) of all GC samples. The association between clinicopathological characteristics and miR-198 expression was summarized in Table 1. We found that miR-198 level was associated with tumor size (*p* = 0.009), tumor depth (*p* = 0.005), lymph node metastasis (*p* = 0.03), and clinical stage (*p* = 0.02). However, we did not find any significant correlation between miR-198 levels and other clinicopathological features, such as patient’s gender, age, Lauren type, and cancer differentiation.

### Prognostic values of miR-198 expression in GC patients

**Fig. 2:**
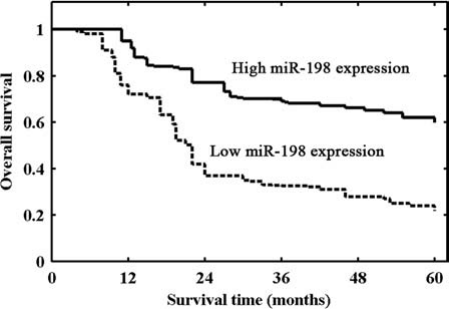
Kaplan-Meier survival curves of 106 gastric cancer patients based on miR-198 expression status. Patients in the low expression group had significantly poorer prognosis than those the in high expression group (*p* < 0.001, log-rank test)

We further evaluated the association of miR-198 expression level with survival of GC patients. Survival analysis indicated that patients in the high miR-198 expression group had better 5-year overall survival than those in the low miR-198 expression group (*p* < 0.001, Fig. 2). Univariate analysis revealed that miR-198 expression (*p* < 0.001), tumor size (*p* = 0.006), tumor depth (*p* = 0.015), lymph node metastasis (*p* = 0.028), and TNM stage (*p* < 0.001) were prognostic factors for patient’s overall survival (Table 2). Multivariate analysis confirmed low miR-198 expression (*p* = 0.015, RR = 2.52) as an unfavorable prognostic indicator independent of other clinicopathological factors, including depth of infiltration (*p* = 0.003, HR = 3.17), lymph node status (*p* = 0.012, HR = 2.79), and TNM stage (*p* = 0.008, HR = 2.95; Table 2).

**Table 2:** Univariate and multivariate analysis of overall survival in 106 gastric cancer patients

Variables	Univariate analysis		Multivariate analysis	
	RR	*p* value	RR	*p* value
Age	0.96	0.228	1.47	0.098
Gender	1.43	0.135	0.98	0.275
Differentiation	1.57	0.096	1.56	0.079
Lauren type	1.35	0.144	1.19	0.212
Tumor size	3.11	0.006	1.55	0.084
Invasion depth	2.87	0.015	3.17	0.003
Lymphatic metastasis	2.59	0.028	2.79	0.012
TNM stage	3.97	<0.001	2.95	0.008
miR-198 expression	3.82	<0.001	2.52	0.015

## Discussion

Identifying novel molecules that take part in GC formation and progression may be helpful for improving the diagnosis, prevention and treatment of this disease. The relationship between miRNAs and tumors has currently become one of the focuses of cancer studies. Abnormal expression of several miRNAs has been reported in GC. For example, miR-1271 was downregulated in GC tissues and correlated with tumor size, lymph node metastasis, and tumor stage [25]. High miR-221 expression and low miR-34a expression are associated with poor prognosis in GC patients [26, 27]. Circulating miR-18a in plasma could discriminate GC patients from healthy controls with a clinically satisfactory degree of sensitivity and specificity [28]. Functional analysis indicated that ectopic miR-874 expression suppressed the growth, migration, invasion, and tumorigenicity of GC cells, whereas miR-874 knockdown promoted these phenotypes [29]. miR-223 and miR-20a could promote cisplatin resistance of GC cells via regulating cell apoptosis [30, 31]. These findings suggested that miRNAs might play important roles in GC initiation and development and have a great potential for clinical application.

In the current study, we found that miR-198 was downregulated in human GC tissues compared with noncancerous tissues. We also found that decreased miR-198 expression was significantly correlated with aggressive clinicopathological features. Moreover, Kaplan-Meier analysis showed that GC patients with low miR-198 expression tend to have shorter overall survival. The multivariate analysis confirmed low miR-198 expression as an independent predictor of poor survival. To the author’s knowledge, this is the first study to analyze the expression and clinical significance of miR-198 in GC.

Previous research has demonstrated the tumor-suppressive functions of miR-198 in several human cancers. Decreased miR-198 expression in colorectal cancer showed a significant association with histological grade, T stage, lymph node invasion, and AJCC stage [18]. Overexpression of miR-198 in colorectal cancer cell lines inhibited cell proliferation, invasion, and migration by targeting fucosyl transferase 8 in vitro. In vivo, restoration of miR-198 significantly inhibited xenograft growth and invasion in nude mice. In pancreatic cancer, reconstitution of miR-198 resulted in reduced tumor growth and metastasis through direct targeting MSLN, PBX-1, and VCP [20]. Low miR-198 levels in pancreatic cancer tissue samples predicted shorter overall survival. In addition, Yang et al. reported that miR-198 inhibited proliferation and induced apoptosis of A549 lung cancer cells via targeting FGFR1 [17]. Tan et al. found that miR-198 inhibited hepatocellular carcinoma cell invasion and migration by targeting the HGF/c-MET pathway [19]. Taken together, these research indicated that loss of miR-198 might contribute to cancer formation and progression. However, the complex molecular mechanisms underlying low miR-198 expression in human cancers and its function are still incompletely known. More studies should be applied to clarify the precise mechanisms by which miR-198 exerts antitumor activity.

## Conclusions

In conclusion, our study showed that miR-198 was downregulated in GC tissues and correlated with aggressive clinicopathological features. Furthermore, low miR-198 expression was an important indicator for unfavorable prognosis of GC patients. Due to the limited sample size in our study, further prospective analysis with a large number of tumor samples is worth doing to verify these conclusions.
