# Expression of Concern to: Decreased miR-198 expression and its prognostic significance in human gastric cancer

**DOI:** 10.1186/s12957-019-1643-3

**Published:** 2019-06-13

**Authors:** Zhigang Cui, Xin Zheng, Di Kong

**Affiliations:** Department of Oncology, Tianjin Nankai Hospital, No.6 of Changjiang Road, Nankai District, Tianjin, 300100 People’s Republic of China

**Expression of Concern to: World J Surg Oncol**

**https://doi.org/10.1186/s12957-016-0784-x**

The Editor-in-Chief is issuing an editorial expression of concern to alert readers that this article [1] contains substantial overlap with articles published within a close time frame [2–5]. None of the authors have responded to any correspondence from the editor or publisher about this editorial expression of concern.
